# Hospital admission and the occurrence of delirium in older adults with physical frailty: cross-sectional study^*^

**DOI:** 10.1590/1980-220X-REEUSP-2023-0156en

**Published:** 2023-12-15

**Authors:** João Alberto Martins Rodrigues, Maria Helena Lenardt, Clovis Cechinel, Elaine Drehmer de Almeida Cruz, Audrey Tieko Tsunoda, Tatiane Prette Kuznier

**Affiliations:** 1 Universidade Federal do Paraná, Programa de Pós-graduação em Enfermagem, Curitiba, PR, Brazil.; 2 Pontifícia Universidade Católica do Paraná, Programa de Pós-graduação em Tecnologias em Saúde, Curitiba, PR, Brazil.

**Keywords:** Frail Elderly, Hospitalization, Delirium, Cross-sectional Studies, Anciano Frágil, Hospitalización, Delirio, Estudios Transversales, Idoso Fragilizado, Hospitalização, Delírio, Estudos Transversais

## Abstract

**Objective:**

To analyze the relationship between hospitalization and the occurrence of delirium in older adults with physical frailty.

**Method:**

Cross-sectional study carried out in a public hospital in southern Brazil. Hospitalized older adults aged ≥ 60 years participated. Sociodemographic and clinical data were collected, physical frailty phenotype tests were performed and the Confusion Assessment Method was used. Descriptive analyzes were carried out and odds ratio values were estimated for the frailty and delirium variables.

**Results:**

Of the 320 older adults evaluated, 21.14% presented delirium, 49% were identified as pre-frail and 36.2% as frail. Of those affected by delirium, 71.6% were classified as frail and 28.3% as pre-frail (p < 0.001). An association was observed between the occurrence of delirium and frailty (OR 1.22; 95% CI 1.07 to 1.38), age ≥ 80 years (OR 1.14; 95% CI 1.01 to 1.32), epilepsy (OR 1.38; 95% CI 1.09 to 1.76), dementia (OR 1.58; 95% CI 1.37 to 1.82), and history of stroke (OR 1.14; 95% CI 1.03 to 1.26).

**Conclusion:**

There was a high frequency of pre-frail and frail older adults, and the occurrence of delirium in frail was significantly higher. Special attention should be paid to frail older adults to prevent the occurrence of delirium during hospitalization.

## RESUMO

**Objetivo:**

Analisar a relação entre hospitalização e ocorrência de *delirium* em idosos na condição de fragilidade física.

**Método:**

Estudo transversal desenvolvido em hospital público no Sul do Brasil. Participaram idosos hospitalizados, idade ≥ 60 anos. Foram coletados dados sociodemográficos e clínicos, e feitos testes do fenótipo da fragilidade física, bem como o *Confusion Assessment Method*. Realizaram-se análises descritivas e estimaram-se as *odds ratio* para as variáveis fragilidade e *delirium*.

**Resultados:**

Dos 320 idosos avaliados, 21,14% apresentaram *delirium,* 49% foram identificados como pré-frágeis e 36,2% frágeis. Dos idosos acometidos por *delirium,* 71,6% foram classificados como frágeis e 28,3% pré-frágeis (*p*< 0,001). Observou-se associação entre ocorrência de *delirium* e fragilidade (OR 1,22; IC 95% 1,07 a 1,38), idade ≥ 80 anos (OR 1,14; IC 95% 1,01 a 1,32), epilepsia (OR 1,38; IC 95% 1,09 a 1,76), demência (OR 1,58; IC 95% 1,37 a 1,82), e história de acidente vascular encefálico (OR 1,14; IC 95% 1,03 a 1,26).

**Conclusão:**

Observou-se alta frequência de idosos pré-frágeis e frágeis e ocorrência de *delirium* expressivamente maior nos frágeis. Atenção especial deve ser empregada a idosos frágeis para prevenir a ocorrência de *delirium* durante a hospitalização.

## RESUMEN

**Objetivo:**

Analizar la relación entre la hospitalización y la aparición de delirio en adultos mayores con fragilidad física.

**Método:**

Estudio transversal realizado en un hospital público del sur de Brasil. Participaron adultos mayores hospitalizados con edad ≥ 60 años. Se recogieron datos sociodemográficos y clínicos, se realizaron pruebas de fenotipo de fragilidad física y se utilizó el *Confusion Assessment Method*. Se realizaron análisis descriptivos y se estimaron los odds ratio para las variables fragilidad y delirio.

**Resultados:**

De los 320 adultos mayores evaluados, el 21,14% presentó delirio, el 49% fueron identificados como prefrágiles y el 36,2% como frágiles. De los adultos mayores afectados por delirio, el 71,6% fueron clasificados como frágiles y el 28,3% como prefrágiles (p < 0,001). Se observó asociación entre la aparición de delirio y fragilidad (OR 1,22; IC 95% 1,07 a 1,38), edad ≥ 80 años (OR 1,14; IC 95% 1,01 a 1,32), epilepsia (OR 1,38; IC 95% 1,09 a 1,76), demencia (OR 1,58; IC del 95%: 1,37 a 1,82) y antecedentes de accidente cerebrovascular (OR 1,14; IC del 95%: 1,03 a 1,26).

**Conclusión:**

Hubo una alta frecuencia de adultos mayores prefrágiles y frágiles y la aparición de delirio en los frágiles fue significativamente mayor. Se debe prestar especial atención a los adultos mayores frágiles para prevenir la aparición de delirio durante la hospitalización.

## INTRODUCTION

Older adults are regular users of health services, with an increasing frequency with advancing age^(1)^. When it comes to hospital admission, older adults are twice as likely to require hospitalization compared to middle-aged adults^(2)^, a condition that can trigger undesirable and preventable outcomes, such as delirium.

Delirium is characterized by global cognitive dysfunction, attention deficit and altered state of consciousness, with an acute onset and fluctuating course^(3)^. Severe and often fatal in older adults, it is associated with longer length of hospital stay, long-term functional and cognitive impairment, and increased mortality^(4)^. As it is underdiagnosed by healthcare professionals, it is associated with a poor short- and long-term prognosis and high costs for the healthcare system^(5)^.

This syndrome is among the most common in the population of older adults, who present several risk factors for its development^(6)^. It can be triggered by an isolated factor, although it is commonly interrelated to other conditions that cause, aggravate or increase its risk. The effects of risk factors for delirium seem to be cumulative, among which advanced age, low body mass index (BMI), visual impairment, dementia and hypertension stand out^(7)^.

Because of its association with negative outcomes, physical frailty is an important concept related to healthcare. This is a clinical condition of greater vulnerability when the individual is exposed to internal and external stressors. This syndrome stands out as one of the main contributors to functional decline and early mortality in older adults^(8)^.

The hospitalization event is directly related to the frailty process in older adults, which is associated with a higher mortality rate, advanced age, readmission and transfers to Long-Term Care Facilities for Older Adults^(9)^. The variation in prevalence of frailty among hospitalized patients is wide, and rates range between 24.7% and 80%^(10)^.

In this sense, information on hospital admissions in Brazil, as well as the major causes of mortality and corresponding sociodemographic variables can be obtained from secondary sources of health information systems. However, associations between the variables of interest, frailty and delirium in hospitalized older adults are lacking^(11)^, as well as specific national studies on the effects of hospital admission on frailty and the occurrence of delirium in older adults.

Frailty and delirium are two of the most complex management problems among hospitalized older adults. A systematic literature review with meta-analysis conducted in Curitiba (Brazil) evaluated 26 articles, totaling a sample of 13,203 older adults with the aim of synthesizing evidence of the relationship between frailty and delirium in hospitalized older adults. A prevalence of 34% of frailty and 21% of delirium was observed, and frailty was an independent risk factor for the development of delirium, with an increased chance of 66% compared to non-frail older adults^(12)^.

The association between these two factors can have important clinical consequences. The presence of frailty can lead to the risk of delirium and this can worsen a pre-existing state of frailty, in addition to hindering the functional recovery of hospitalized older adults, creating a greater risk of institutionalization and disability^(13)^.

Given the above, the aim of this study was to analyze the relationship between hospitalization and the occurrence of delirium in older adults with physical frailty.

## METHOD

### Study Design

This is a quantitative, cross-sectional study derived from a master’s thesis in which the prevalence of physical frailty and delirium in hospitalized older adults was identified and the relationship between variables was analyzed.

### Location

The study was carried out in a public hospital in southern Brazil, which focuses on low and medium complexity outpatient, hospital and home care. The institution offers the Unified Health System (SUS) 143 beds, distributed in 60 ICU beds, six Observation beds, three Emergency beds and 74 beds in Hospitalization Units.

### Population, Inclusion and Exclusion Criteria

Older adults aged ≥60 years constituted the study population. The inclusion criteria were age ≥60 years and being hospitalized for clinical and/or surgical treatment during the data collection period. The exclusion criteria were presenting a critical condition, such as the need for non-invasive ventilation, imminent death, hypotension and patients with indication and awaiting transfer to the ICU, which made it impossible to apply the tests; in addition to recommendation for isolation by droplets or aerosols.

When the older adults presented cognitive impairment and/or significant communication deficits, the companion was invited to answer the research questions. The inclusion criteria for the companion were: age ≥18 years and accompanying the older adult for at least three months. Companions aged ≥60 years should have cognitive capacity identified by the MMSE, used to determine their status as a respondent, according to cutoff points adopted by educational level^(14,15)^. Having communication difficulties (speech and/or hearing) was an exclusion criterion.

Of the 320 older adults analyzed, 117 were alone and able to answer the questionnaire questions and 203 were accompanied. Among the 203 older adults with a companion, 57 were able to respond to the questionnaire (according to the MMSE). The companions of the remaining 146 older adults answered the questionnaire questions.

### Sample Calculation and Data Collection

The time frame of 2019 was used to calculate the sample size. During the analyzed period, the study hospital had 7,254 admissions, of which 4,146 were older adults aged ≥60 years. A 30% prevalence of delirium was considered in the population of hospitalized older adults^(5,12)^, obtaining a sample size of at least 300 individuals.

Data collection was carried out from March to July 2022. The following were established as independent variables: sociodemographic (sex, age, education, color, with whom one resides, professional status, income, type and reason for hospitalization); clinical (systemic arterial hypertension, diabetes mellitus, Chronic Obstructive Pulmonary Disease/COPD, Chronic Kidney Disease/CKD, epilepsy, congestive heart failure, stroke, dementia, polypharmacy and number of medications); laboratory (sodium, potassium, leukocytes, C-Reactive Protein/CRP); and physical frailty. The presence of delirium corresponded to the dependent variable.

Physical frailty was assessed in the first 48 hours of hospital admission, considering the five components of Fried’s phenotype; unintentional weight loss, slow walking speed, weakness or poor handgrip strength, self-reported exhaustion and low physical activity^(16)^.

Delirium was assessed within the first 48 hours of hospital admission using the Confusion Assessment Method^(17)^ validated for Brazilian Portuguese^(18)^. Four cardinal characteristics that allow the distinction of delirium from other types of cognitive impairment were analyzed: A) acute onset and fluctuating evolution, B) attention deficit, C) disorganized thinking and D) change in the level of consciousness. The diagnosis of delirium was given by the presence of criteria A, B and C or D.

### Data Analysis and Processing

To reduce the risk of bias, the representative sample of the population was defined based on the sample calculation. A 30% prevalence of delirium in the hospitalized population of older adults was considered in the calculation^(5,12,19)^, bringing statistical validity to the information generated in the analyzes and inferring its validity for the population. Some parameters were established in advance; 95% confidence level and 5% sampling error.

The team of examiners was trained in the use of data collection instruments, which were standardized for a uniform and consistent collection of data in a private environment, in an attempt to avoid interference, noise and embarrassment for participants.

Descriptive statistics techniques were used to identify the sociodemographic characteristics. The number of individuals per characteristic and the respective proportions and confidence intervals were recorded in descriptive tables. Overlapping confidence intervals indicated there was no statistical difference between proportions.

The equality hypothesis test was used to compare the proportions of responses and assess the difference between the proportion of individuals diagnosed with delirium among patients classified as pre-frail or frail. In situations where frequentist statistics did not produce good estimates, Bayesian statistics was used.

Crude estimates of odds ratios (OR) relating to the association between the variables of interest and outcomes were obtained, as well as adjusted odds ratios for the complete model and the respective 95% confidence intervals (95% CI).

### Ethical Aspects

This study is a subproject of a matrix study approved by the Human Research Ethics Committee of the Health Sciences Department of the Universidade Federal do Paraná (UFPR, Brazil) under opinion number 4.985.540 of September 2021, and by the Research Ethics Committee from the Municipal Health Department (SMS, Curitiba, Brazil), opinion number 5.055.260 of October 2021. The informed consent instrument was signed by the older adult or responsible companion before the beginning of the study according to Resolution 466/12.

## RESULTS

The sample of 320 older adults was composed of 59.4% of women, 40.9% aged ≥ 80 years and 66.2% with low schooling. White people predominated (70.60%), 42.2% were widowed, 76.2% living with their spouse, children, siblings or other relative, 14.7% living alone and 87.2% retired or pensioners. Of the older adults who reported income, 48.5% received from zero to one minimum wage. In relation to family income and excluding patients who did not want to inform it, 40.6% received one to three minimum wages. During hospitalization, 63.4% of older adults were accompanied, of which 43.8% by their spouse or child and 7% by professional caregivers. Clinical hospitalizations (92.2%) and diagnoses of pneumonia (17.8%) and urinary tract infection (16.20%) predominated. Other pathologies led to hospitalization, such as ischemic stroke outside the thrombolysis window (9.7%), cholecystitis and acute pancreatitis (5.6%), congestive heart failure (4.1%), hematuria and prostatitis due to benign hyperplasia of prostate (2.2%), deep vein thrombosis, consumptive syndrome, among others (Table 1).

**Table 1:** Distribution and confidence interval of the sociodemographic characteristics of older adults in the sample. Curitiba, PR, Brazil, 2022.

	TOTAL	
	n = 320	95%CI^*^ (SD)^†^
**Sex**		
Female	190	59.4% (53.9–64.6)
Male	130	40.6% (35.4–46.1)
**Age**		
60–70	86	26.9% (22.3–32.0)
70–80	103	32.2% (27.3–37.5)
80 or more	131	40.9% (35.7–46.4)
**Education**		
Illiterate	59	18.4% (14.6–23.1)
Literate (did not attend school)	37	11.6% (8.5–15.5)
Incomplete primary school	116	36.2% (31.2–41.7)
Primary school	65	20.3% (16.3–25.1)
Incomplete secondary school	10	3.1% (1.7–5.7)
Secondary school	22	6.9% (4.6–10.2)
Incomplete higher education	2	0.6% (0.2–2.2)
Higher education	9	2.8% (1.5–5.3)
**Color**		
White	226	70.6% (65.4–75.3)
Brown/mixed race	68	21.2% (17.1–26.1)
Black	23	7.2% (4.8–10.6)
Yellow/Asian	3	0.9% (0.3–2.7)
**Marital status**		
Single	17	5.3% (3.3–8.3)
Married	131	40.9% (35.7–46.4)
Divorced	37	11.6% (8.5–15.5)
Widowed	135	42.2% (36.9–47.7)
**Residing with**		
Spouse and/or children	235	73.4% (68.3–78.0)
Parents and/or siblings	9	2.8% (1.5–5.3)
Alone	47	14.7% (11.2–19.0)
LTCF	12	3.8% (2.2–6.4)
Others	17	5.3% (3.3–8.3)
**Professional situation**		
Working	18	5.6% (3.6–8.7)
Retiree	214	66.9% (61.5–71.8)
Pensioner/on benefits Social assistance	65	20.3% (16.3–25.1)
Home worker	11	3.4% (1.9–6.0)
Unemployed	12	3.8% (2.2–6.4)
**Older adult income (minimum wage)**		
No income	17	5.3% (3.3–8.3)
0–1	127	39.7% (34.5–45.1)
1–3	120	37.5% (32.4–42.9)
3–5	27	8.4% (5.9–12.0)
5–10	5	1.6% (0.7–3.6)
More than 10	1	0.3% (0.1–1.7)
Did not inform	23	7.2% (4.8–10.6)
**Family income (minimum wage)**		
No income	1	0.3% (0.1–1.7)
0–1	35	10.9% (8.0–14.8)
1–3	89	27.8% (23.2–33.0)
3–5	75	23.4% (19.1–28.4)
5–10	16	5% (3.1–8.0)
More than 10	3	0.9% (0.3–2.7)
Did not inform	101	31.6% (26.7–36.8)
**Companion’s degree of kinship**		
Spouse and/or children	140	43.8% (38.4–49.2)
Parents and/or siblings	3	0.9% (0.3–2.7)
Alone	117	36.6% (31.5–42.0)
Professional caregiver	7	2.2% (1.1–4.4)
Others	53	16.6% (12.9–21.0)
**Type of current hospitalization**		
Clinical	295	92.2% (88.7–94.7)
Surgical	25	7.8% (5.3–11.3)
**Reason for current hospitalization**		
Pneumonia	57	17.8% (14.0–22.4)
Urinary tract infection	52	16.2% (12.6–20.7)
Stroke	31	9.7% (6.9–13.4)
Others	180	56.2% (50.8–61.6)

^*^95% CI – 95% Confidence Interval;

^†^SD – Standard Deviation; LTCF – Long-Term Care Facility.

**NOTE:** Official Gazette of the Union published on 31 December, 2021 – Provisional Measure Number. 1.091 establishes that as of 1 January, 2022, the minimum wage is R$1,212.00.

**SOURCE:** The authors (2022).

In the first 48 hours of hospital admission, there was a predominance of pre-frail older adults (n = 157; 49%), followed by frail (n = 116; 36.2%) and non-frail (n = 44; 13.7%). Within the first 48 hours of hospital admission, 67 (21.1%) patients were diagnosed with delirium. Of these, 71.6% were classified as frail and 28.3% were pre-frail (p < 0.001). During hospitalization, 13.43% of older adults developed delirium. Among the older adults who developed delirium, 4.54% were classified as non-frail, 10.94% as pre-frail, and 37.68% as frail.

There was an association between age over 80 years and the presence of delirium (OR 1.22; 95% CI 1.08 to 1.37); delirium and epilepsy (OR 1.35; 95% CI 1.10 to 1.66); and dementia (OR 1.63; 95% CI 1.46 to 1.81) and stroke (OR 1.14; 95% CI 1.03 to 1.26) (Table 2)

**Table 2:** Association between the presence of delirium and sociodemographic and clinical variables. Curitiba, PR, Brazil, 2022.

Variables	OR^*^	95% CredI^†^	Adjusted OR^‡^	95% CredI^†^
**Sex**				
Female	1.00	Ref.	1,00	Ref.
Male	0.98	0.89; 1.08	0.98	0.89; 1.08
**Color**				
White	1.00	Ref.	1,00	Ref.
Non white	0.97	0.88; 1.08	0.97	0.88; 1.07
**Age**				
60–70	1.00	Ref.	1,00	Ref.
70–80	1.01	0.90; 1.13	0.98	0.87; 1.11
**80 or over**	**1.31**	**1.17; 1.46**	**1.22**	**1.08; 1.37**
**Schooling**				
Primary	1.00	Ref.	1,00	Ref.
Secondary	0.94	0.81; 1.10	1.03	0.89; 1.19
Higher Education	0.93	0.70; 1.22	0.94	0.72; 1.22
**Systemic Arterial Hypertension**				
No	1.00	Ref.	1,00	Ref.
Yes	0.90	0.81; 0.99	0.95	0.87; 1.05
**Diabetes mellitus**				
No	1.00	Ref.	1.00	Ref.
Yes	0.92	0.83; 1.01	1.01	0.92; 1.10
**Chronic obstructive pulmonary disease**				
No	1.00	Ref.	1.00	Ref.
Yes	0.94	0.83; 1.06	0.95	0.85; 1.06
**Chronic kidney disease**				
No	1.00	Ref.	1.00	Ref.
Yes	0.91	0.78; 1.06	0.92	0.81; 1.06
**Epilepsy**				
No	1.00	Ref.	1.00	Ref.
**Yes**	**1.35**	**1.06; 1.71**	**1.35**	**1.10; 1.66**
**Congestive heart failure**				
No	1.00	Ref.	1.00	Ref.
Yes	0.93	0.83; 1.05	0.97	0.87; 1.08
**Dementia**				
No	1.00	Ref.	1.00	Ref.
**Yes**	**1.68**	**1.52; 1.85**	**1.63**	**1.46; 1.81**
**Previous stroke**				
No	1.00	Ref.	1.00	Ref.
**Yes**	**1.23**	**1.10; 1.37**	**1.14**	**1.03; 1.26**

^*^OR – Odds Ratio;

^†^95% CredI – 95% Credible interval;

^‡^Adjusted OR – Adjusted Odds Ratio.

**SOURCE:** The authors (2022).

Of the laboratory variables, there was an association between delirium and increased CRP values. The chance of an older adult with a CRP above the reference values used by the laboratory being diagnosed with delirium is 14% greater than that of an older adult with a normal CRP (below 0.5ng/dL) (Table 3).

**Table 3:** Association between the presence of delirium and laboratory variables, Curitiba, PR, Brazil, 2022.

Variables	OR^*^	95%CI^†^	Adjusted OR^‡^	95%CI^†^	p Value^§^
Intercept			0.12	0.02; 0.54	0.0092
**Sodium**					
Normal	1.00	Ref.	1.00	Ref.	
Below normal	0.93	0.83; 1.04	0.92	0.82; 1.03	0.2765
**Potassium**					
Below normal	0.98	0.40; 2.21	0.90	0.33; 2.23	0.8258
Normal	1.00	Ref.	1.00	Ref.	
Above normal	1.67	0.61; 4.18	1.69	0.54; 4.86	0.3471
**Leukocytes**					
Below normal	1.06	0.05; 7.49	0.77	0.04; 6.04	0.8248
Normal	1.00	Ref.	1.00	Ref.	
Above normal	1.50	0.82; 2.71	0.95	0.46; 1.93	0.8961
**PCR**					
Normal	1.00	Ref.	1.00	Ref.	
**Above normal**	**1.17**	**1.02; 1.33**	**1.14**	**1.02; 1.30**	**0.0495**

^*^OR – Odds Ratio;

^†^95%CI – 95% Confidence Interval;

^‡^Adjusted OR – Adjusted Odds Ratio;

^§^p-value – obtained via Wald test.

**NOTE:** reference values according to collection laboratory: Sodium – Adults: 138–145 mmol/L; >90 years: 132–146 mmol/L; Potassium: 3.5–5.1 mol/L; Leukocytes: 3,800–11,000/mm^3^; C-Reactive Protein: less than 0.5ng/dL.

**SOURCE:** The authors (2022).

Upon hospital admission, 30.3% of patients had hyponatremia, 28.1% had acute renal failure, 71.9% had CRP above reference values, 71.2% had a hemoglobin level below normal and 34.7% leukocytosis. Polypharmacy was evidenced in 40.9%, and 7.2% were considered as hyper polypharmacy with a mean (SD) use of 4.57 (3.06) medications (Table 4).

**Table 4:** Distribution and confidence interval of laboratory characteristics and polypharmacy of older adults in the sample. Curitiba, PR, Brazil, 2022.

	TOTAL	
	n = 320	95% CI^*^ (SD)^†^
**Sodium**		
Normal	205	64.1% (60.3–70.8)
Below normal	97	30.3% (26.2–36.4)
Above normal	10	3.1% (1.8–5.8)
Not collected	8	
**Acute kidney failure**		
Yes	90	28.1% (23.8–33.7)
No	226	70.6% (66.3–76.2)
Not collected	4	
**CRP**		
Normal	47	14.7% (13.0–21.8)
Above normal	230	71.9% (78.2–87.0)
Not collected	43	
**Hemoglobin**		
Normal	82	25.6% (21.6–31.3)
Below normal	228	71.2% (67.7–77.5)
Above normal	3	0.9% (0.3–2.8)
Not collected	7	
**Potassium**		
Normal	245	76.6% (73.4–82.5)
Below normal	42	13.1% (10.1–17.6)
Above normal	26	8.1% (5.7–11.9)
Not collected	7	
**Leukocytes**		
Normal	198	61.9% (57.6–68.2)
Below normal	5	1.6% (0.7–3.7)
Above normal	111	34.7% (30.3–40.8)
Not collected	6	
**Polypharmacy**		
Normal	166	51.9% (46.4–57.3)
Polypharmacy	131	40.9% (35.7–46.4)
Hyper polypharmacy	23	7.2% (4.8–10.6)
**Number of medicines for home use**		
Mean (SD)	4.57 (3.06)	
Median [Min, Max]	4.00 [0. 16.0]	

^*^IC 95% CI – 95%Confidence Interval;

^†^SD – Standard Deviation.

**NOTE:** reference values according to collection laboratory; Sodium – Adults: 138–145 mmol/L; >90 years: 132–146 mmol/L; Hemoglobin: 13.5–17.5g/dL; Leukocytes: 3,800–11,000/mm^3^; C-Reactive Protein: less than 0.5ng/dL.

**SOURCE:** The authors (2022).

Table 5 shows the distribution of absolute and relative frequency of diseases. Arterial hypertension affects 71.2% of older adults, 34.4% have diabetes mellitus, 20.9% dementia, 20% have already been affected by a stroke and 19.7% have hypothyroidism and/or dyslipidemia.

**Table 5:** Distribution of the absolute and relative frequency of diseases present in older adults in the sample. Curitiba, PR, Brazil, 2022.

	Total	
	(n = 320)	(%)
Arterial hypertension	228	71.2%
Diabetes mellitus	110	34.4%
Dementia	67	20.9%
Brain stroke	64	20%
Hypothyroidism	63	19.7%
Dyslipidemia	63	19.7%
Congestive heart failure	59	18.4%
COPD	51	15.9%
Ischemic heart disease	37	11.5%
Depression	35	10.1%
Chronic kidney disease	23	7.2%
Parkinson’s disease	13	4%
Epilepsy	12	3.7%

**SOURCE:** The authors (2022).

## DISCUSSION

In the present study, there was an association between frailty and delirium (OR 1.22; 95% CI 1.07 to 1.38). In a retrospective cohort study of 218 older adults, mean age of 71.54 years (SD 9.53 years), conducted in the United States of America (USA), an association between frailty and delirium was found (non-frail: 3. 6%; pre-frail: 20.9% and frail: 29.3%, p = 0.001). After adjustment for sociodemographic data, pre-frail (adjusted OR 5.64; 95% CI 1.23 to 25.99) and frail (adjusted OR 6.80; 95% CI 1.38 to 33.45) patients were independently associated with delirium^(19)^.

Approximately half of the sample of hospitalized older adults presented a pre-frailty condition, while 36.2% showed frailty. In a systematic review of the literature with 96 studies, totaling a sample of 467,779 hospitalized older adults aged ≥ 65 years, the distribution of frailty conditions was similar to that of the present study. There was a predominance of pre-frailty, 47.4% (95%CI 43.7 to 51.1%), followed by frailty, 25.8% (95% CI 20 to 29.6%)^(20)^.

The Cardiovascular Health Study (CHS) was conducted with 5,201 older adults aged ≥65 years to operationalize the physical frailty phenotype. Similar percentages of older adults in pre-frail (46.4%) and non-frail (46.7%) conditions were identified^(16)^. These results corresponded to approximately one fifth of the frequency observed in the present study, even though the distribution among pre-frail patients showed similar values. In this study, hospitalized older adults ≥ 80 years old (63% CI 95% 54.1 to 71.2) predominated.

High rates of frailty highlight the need for screening this physical condition both in the community and in the hospital setting, providing support for care management in the older adult population. Frailty is a reversible condition, and there must be a comprehensive management plan that encompasses polypharmacy, management of sarcopenia and treatable causes of weight loss and fatigue, as well as resistance exercise, assessment of nutrition and oral health^(8)^. According to authors, the interventions do not require high technological complexity or high-cost therapies, but integrated actions by health professionals^(21)^.

Delirium was identified in just over a fifth of the older adults in this sample within the first 48 hours of hospital admission. Most older adults affected by delirium were frail, followed by pre-frail. In view of this, frail older adults were more likely to develop delirium, and the hospital environment by itself or by acute pathologies that lead older adults to hospitalization, is a triggering factor. These results are corroborated by a systematic review of the literature that aimed to estimate the prevalence and summarize the evidence of the relationship between frailty and delirium in hospitalized older adults. The analysis included 26 articles, totaling a sample of 13,203 older adults, and a frequency of 21% of delirium was observed (95% CI 0.17 to 0.25, p < 0.010)^(12)^.

There was an association between age ≥ 80 years and the occurrence of delirium in hospitalized older adults (OR 1.22; 95% ICred 1.08 to 1.37). This datum converges with that of a Spanish prospective cohort study of 909 older adults ≥ 65 years of age in which the results pointed to advancing age as a risk factor for delirium in hospitalized older adults (HR 1.1; 95% CI 1. 02 to 1.08)^(22)^. In another Spanish cross-sectional study of 356 older adults aged ≥ 65 years, 61.6% of those aged ≥ 86 years presented delirium (p < 0.001). In the age groups of 76–85 years and 65–75 years, the percentages reached 29.6% and 8%, respectively (p < 0.001)^(23)^.

There was an association between delirium and increased CRP levels (OR 1.14; 95% CI 1.02 to 1.30, p = 0.049). This datum corroborates that of a retrospective cross-sectional study conducted in the United Kingdom with 710 patients ≥ 70 years of age, in which there was a strong association between high CRP levels and delirium, independent of other risk factors for it (OR 1.32; CI 95% 1.10 to 1.58, p = 0.003)^(24)^.

Epilepsy was associated with delirium (OR 1.35; 95% CI 1.10 to 1.66) and this relationship was also found in a prospective study of 3,076 older adults > 80 years old admitted to a University Hospital in Switzerland (OR 3.65; 95% CI 2.12 to 6.28, p < 0.001)^(25)^. Post-ictal delirium was described in patients admitted to an intensive care unit^(26)^ in a scoping review conducted in Germany that included an analysis of 33 studies and a total sample of 3,122 older adults. In addition to pointing out the association between delirium and the ictal and post-ictal period, in this review, sporadic epileptiform discharges were identified in 20% of patients affected by delirium^(27)^.

In this sample, an association was observed between dementia and delirium (adjusted OR 1.63; 95% CI 1.46 to 1.81), and the chance of an older adult with dementia being diagnosed with delirium was 63% greater than that of an older adult without dementia. This finding confirms that of a systematic review conducted in China that analyzed 22 studies (5,125 patients) and identified an association between dementia and delirium (OR 5.06; 95% CI 2.32 to 11.04)^(28)^. In a prospective cohort study of 3,076 older adults aged ≥ 80 years, a relationship between dementia and delirium (OR 15.6; 95% CI 10.17 to 23.91, p < 0.001) was found. The frequency of delirium reached 41.8% of the older adults over 80 years of age, the hospitalization time was twice as long (p < 0.001), dependence after discharge was greater (OR 2.2; 95% CI 1.73 to 2.8, p < 0.001), as well as mortality (OR 24.88; 95% CI 13.75 to 45.03; p < 0.001)^(25)^.

The association between delirium and cognitive decline has been studied. A systematic review of the literature with meta-analysis of 24 studies involving 10,549 older adults with a mean age of 75.4 (7.6) years showed a strong association between delirium and long-term cognitive decline both in clinical and surgical patients (OR 2. 30; 95% CI 1.85 to 2.86)^(29)^.

Furthermore, there was an association between delirium and stroke (OR 1.14; 95% CI 1.03 to 1.26). The relationship was also found in a systematic review conducted in the USA with 34 studies comprising a sample of 15,650 older adults ≥ 60 years old. After meta-analysis, the odds ratio of delirium was estimated at 3.20 (95% CI 1.17 to 8.75) among patients with a history of stroke^(30)^.

The importance of screening for physical frailty and delirium in the hospitalized older adult population stands out. Physical frailty based on Fried’s phenotype proved to be feasible and important for screening frailty in older adults during hospitalization.

Inferences about the causality of the relationship between variables must be made with caution, as this is a cross-sectional study with sampling results from a single hospital in southern Brazil. Thus, studies evaluating the effects and causal relationship of hospitalization in frail conditions and the occurrence of delirium in older adults should be encouraged, especially in Brazilian older adults.

The limitation of this study was the use of the Minnesota Leisure Time Activities instrument to assess the level of physical activity. As it contains questions related to unusual activities in the reality of the Brazilian population, there may be an impact on the measurement of energy expenditure.

## CONCLUSION

Delirium was identified in just over a fifth of older adults in the sample within the first 48 hours of hospital admission. Most were frail older adults followed by pre-frail. Non-frail older adults did not present delirium, which may show they present less cerebral vulnerability and greater physiological reserve.

Almost half of the sample of hospitalized older adults presented a pre-frailty condition and 36.25% a frailty condition. The prevalence of this condition in the hospital environment demonstrates the importance of its screening and management during hospitalization. Therefore, studies that can reveal the short and long-term consequences of the association of frailty and delirium in hospitalized older adults should be developed.

The identification of frailty raises the possibility of it being a potential therapeutic target in the prevention of delirium in clinical practice. It is expected that the revelation of the impact of frailty on the occurrence of adverse outcomes such as delirium in the hospital environment will help health professionals, especially nursing professionals, to recognize its risks as early as possible and guide actions to prevent its occurrence.
